# Cytomorphologic consideration in malignant ascites with renal cell carcinoma: A report of two cases

**DOI:** 10.4103/1742-6413.62256

**Published:** 2010-04-06

**Authors:** Ruchika Gupta, Sandeep R. Mathur, Venkateswaran K. Iyer, Sudheer Kumar A, Amlesh Seth

**Affiliations:** Departments of Pathology & Urology, All India Institute of Medical Sciences, New Delhi, India

**Keywords:** Peritoneal effusion, renal cell carcinoma, cytology, mesothelial cells

## Abstract

Effusions, especially peritoneal, are seen in less than 2% of patients with renal cell carcinoma (RCC). Since the tumor cells in RCC are bland and nondescript, the involvement of serous effusions is difficult to diagnose. An accurate recognition of malignant effusion and differentiation from reactive mesothelial cells is imperative. A 55-year-old male presented with gradually progressive ascites. Cytospin preparations from ascitic fluid showed reactive mesothelial cells admixed with few smooth-contoured clusters of cells with moderate cytoplasm, vesicular nuclei with prominent nucleolus. He had undergone nephrectomy for papillary RCC two years earlier. Another 36-year-old man underwent left nephrectomy for suspected RCC. Intra-operative ascitic fluid was sent for cytologic examination and showed numerous reactive mesothelial cells along with few clusters of cells with scant to moderate amount of cytoplasm, vesicular nucleus and a small nucleolus. Considering the histomorphology of the primary renal tumor in both cases, a cytologic diagnosis of malignant peritoneal effusion, morphologically compatible with RCC was rendered. RCC, due to its bland cytologic features, is easily overlooked in effusions. In a known patient, the cytopathologist must be extra vigilant to pick up the few cell clusters present in the fluid preparations and differentiate them from reactive mesothelial cells. A close inspection of the cytologic features and comparison with the histopathology of the primary tumor helps in making an accurate diagnosis.

## INTRODUCTION

Renal cell carcinoma (RCC), a common adult renal tumor, rarely involves serous cavities leading to effusions.[1] Due to the bland cytological appearance of cells from RCC, they can easily be confused with mesothelial cells. However, certain subtle cytological features, both architectural and cellular, favor a diagnosis of RCC.[2] The presence of effusions in RCC portends an unfavorable prognosis, and hence an accurate diagnosis is essential.[1] An extensive review of literature yielded only an occasional report of RCC in peritoneal effusion fluids.[1] In these reports also, the subject of diagnosing RCC involvement in serous effusions is not dealt with in great detail.

We describe the cytological features of ascitic fluid in two male patients with RCC (one with papillary RCC and the other with conventional clear cell RCC) and discuss the diagnostic dilemma involved therein.

## CASE REPORTS

### Case 1

A 55-year-old man, a known hypertensive and asthmatic on therapy, presented with a three-month history of progressively increasing abdominal distension associated with dull pain in the abdomen. There was accompanying anorexia and loss of weight. He experienced mild breathlessness while sitting due to the abdominal distension. There was no fever, jaundice, features of gastrointestinal bleed or altered sensorium. Ascitic fluid cytology (at private laboratories) was reported as positive for Mycobacterium tuberculosis on polymerase chain reaction and the patient was started on antitubercular therapy. However, he had deterioration of liver function tests and the therapy was discontinued.

**Figure 1:**
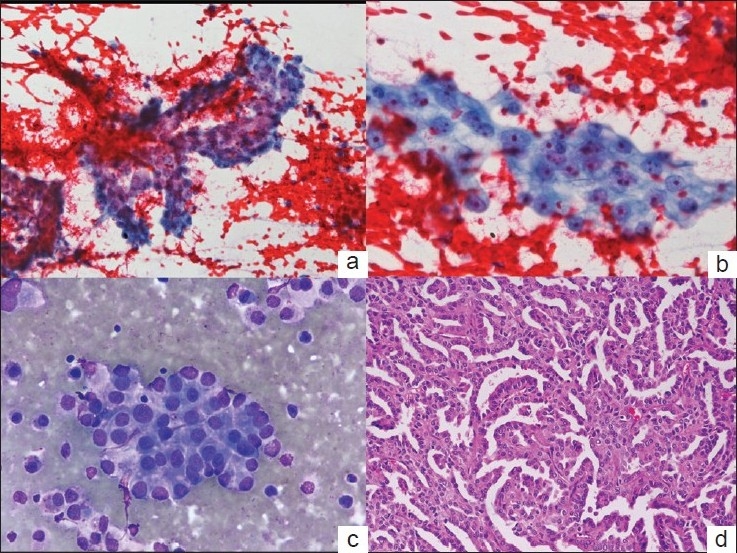
Cytospin smears from Case 1 showing papillary fragments (a. Papanicolaou × 200) of cells with vesicular nuclei and prominent nucleoli (b. Papanicolaou × 400). Focal acinar arrangement is also noted (c. May-Grünwald-Giemsa × 400). Histologic section of the same case showing papillary renal cell carcinoma (d. HandE × 200)

On examination, he had moderate ascites and mild pedal edema. Routine investigations revealed mild elevation of blood urea to 96 mg/dl and serum creatinine to 1.2 mg/dl. Biochemical and cytological analysis of the ascitic fluid showed it to be exudative in nature (protein 4.7 g/dl, total cell count 140/cu.mm.). Cytospin smears prepared from ascitic fluid showed lymphocytes and mesothelial cells in a mildly hemorrhagic background. In addition, occasional clusters and papillary fragments of cells having moderate amount of cytoplasm and central vesicular nucleus with distinct nucleoli were found [Figure 1a–c]. Focal acinar arrangement was noted. The clusters had a smooth outer border. The observed cell clusters resembled mesothelial cells, however ruffled cytoplasmic borders and intercellular “windows” were not identified in these clusters.

Further history was elicited, which revealed that the patient had undergone right radical nephrectomy two years earlier. Pathological examination of the right kidney showed a large 10 ×6 × 4 cm tumor with features of papillary RCC, type I [Figure 1d] confined to the renal capsule without extension to perinephric fat, hilar vessels or ureter. A review of the histologic sections of the renal tumor showed similar features in the cell clusters observed in ascitic fluid smears, and thus, a cytological diagnosis of malignant peritoneal effusion with cells from a RCC was made. Radiologic investigations (ultrasonography and CT scan) did not reveal any metastatic deposit in liver, left kidney or peritoneum. There was ill-defined thickening of the omentum beneath anterior abdominal wall.

### Case 2

**Figure 2:**
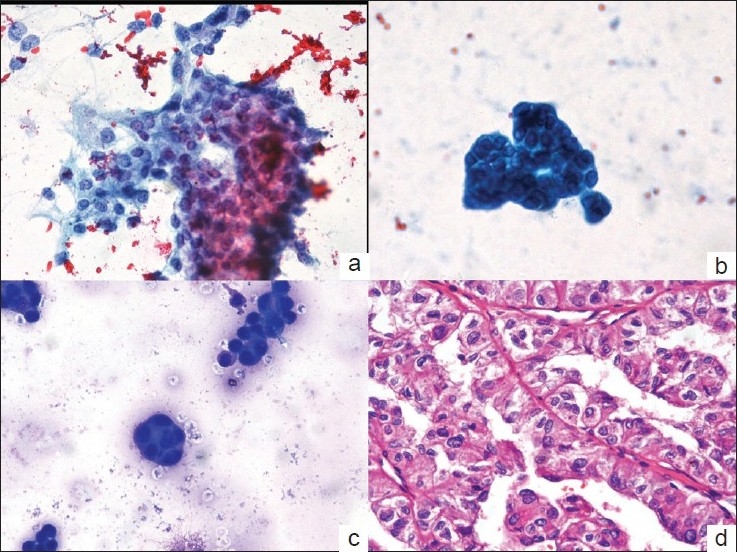
Papanicolaou-stained aspiration smear in Case 2 showing a cohesive cluster of cells with moderate cytoplasm, vesicular nucleus and small nucleoli (a. × 200). Cytospin smear of ascitic fluid of the same patient showing a small cohesive cluster of cells with similar morphology (b. Papanicolaou × 400 and c. May-Grünwald-Giemsa × 200). Photomicrograph of the histologic section showing conventional renal cell carcinoma, Fuhrman nuclear grade 2 (d. HandE × 400)

A 36-year-old man presented with a left flank mass for one year and intermittent hematuria for the past six months. Radiological investigations showed a left renal mass adherent to the descending colon and pancreas. Ultrasound-guided fine needle aspirate (FNA) from the left renal mass showed fragments of tumor cells with moderate amount of cytoplasm, vesicular nucleus, small nucleoli and mild pleomorphism [Figure 2a]. A cytologic diagnosis of RCC was rendered. The patient underwent left nephrectomy with left hemicolectomy, splenectomy and partial pancreatectomy. Intra-operative ascitic fluid was sampled and sent for cytopathologic examination. Smears from the ascitic fluid showed reactive mesothelial cells in a hemorrhagic background. In addition, few small tightly-cohesive clusters of cells [Figure 2b–c], morphologically similar to those seen in the aspiration smears were noted. These clusters did not show intercellular spaces or ‘windows’ or ruffled cytoplasmic borders. Histologic examination of the renal tumor showed features of a conventional (clear cell) RCC, Fuhrman nuclear grade 2 with focal papillary pattern [Figure 2d]. The cells noted in the ascitic fluid were similar to the tumor cells in histologic sections and a final cytologic diagnosis of malignant peritoneal effusion with cells from a RCC was rendered.

## DISCUSSION

RCC is a common tumor seen in adults. Malignant effusions, peritoneal or pleural, are rare in patients with RCC.[1] In large studies of effusion fluids, RCC has been the causative neoplasm in 1-2.2% of malignant pleural effusions.[3] However, benign effusions have also been reported in patients with advanced RCC, and hence, the presence of effusion does not correlate with the stage of primary tumor.[4,5]

The recognition of cells from RCC in effusion fluids may be difficult due to the bland and nondescript nature of tumor cells.[2,6] One series described large tumor cells with abundant foamy and/ or granular cytoplasm as the characteristic feature of RCC in fluids.[7] However, it is a difficult task to differentiate cells of RCC from mesothelial cells due to the bland appearance of the former. Additionally, effusions related to RCC may show reactive mesothelial cells overshadowing the few clusters of malignant cells, as was evident in both our cases. Mesothelial cells may also have foamy appearance due to the intracytoplasmic glycogen vacuoles. However, intracytoplasmic lipid, if detected by Oil Red O, along with immunocytochemistry for CD15, RCC Ma (RCC marker) and CD10 (positive in many RCC) may be helpful in this distinction.[8] Immunostaining for mesothelial markers (calretinin, CK5/6, WT-1 and thrombomodulin) may also assist in the distinction. However, certain antibodies, like BerEP4 and B72.3, which are used frequently in confirmation of metastatic adenocarcinoma in effusion fluids, are not helpful for detection of RCC cells. This is so because the sensitivity of BerEP4 for RCC is quite low.[9] Hence, the panel of immunocytochemistry utilized in differentiation of adenocarcinoma and mesothelial cells (BerEP4, B72.3 and calretinin) would not assist in the same distinction with regards to RCC. In our case, the absence of cytological features of mesothelial cells, namely ruffled cell borders, intercellular gaps or “windows” and the observation of smooth “anatomical” borders of the cell clusters assisted in the diagnosis of metastatic RCC in ascitic fluid in both cases.

Apart from the difficulty in detecting cells from RCC in effusions, the distinction between clear cell and papillary types is also complex, except in cases where papillae are present in the fluids.[1] However, even in such cases where papillae are seen in fluids, these may represent a conglomerate of proliferation spheres. Such proliferation spheres are characteristic of breast carcinoma, but can be seen in other adenocarcinoma as well. Reactive mesothelial cells may also form similar spheres; however scrutiny of the cellular features helps in accurate detection.[10] In addition to the papillae, immunocytochemistry may assist in the differentiation between conventional and papillary RCC. Papillary RCC is more often positive for BerEP4 and less frequently stains with CD10 and vimentin as compared to conventional (clear cell) RCC.[9] Collecting duct type of RCC has a proclivity to involve effusions and shows papillary arrangement of tumor cells with irregular nuclei containing fine chromatin and small nucleoli. Psammoma bodies may be seen in some cases. However, differentiating this morphology from a high-grade papillary RCC may be difficult.[11] In the series by Renshaw *et al*. patients with sarcomatoid RCC developed early but benign effusions, while those with papillary tumors had late-developing malignant effusions. They also reported that though some patients presented with effusions at the time of resection, the effusion never antedated the detection of the primary renal tumor.[1] In one of our cases, the peritoneal effusion was detected two years after the primary diagnosis of papillary RCC. In this patient, no metastatic deposit was found on radiologic investigations. In the other patient, the effusion was seen at the time of resection of the primary tumor.

Malignant effusion in patients with RCC is a rare phenomenon and may be difficult to diagnose cytologically, especially in the presence of reactive mesothelial cells. The cytopathologist must evaluate these effusions carefully to detect the otherwise nondescript cell clusters.

## COMPETING INTEREST STATEMENT BY ALL AUTHORS:

No competing interest to declare by any of the authors.

## AUTHORSHIP STATEMENT BY ALL AUTHORS:

Each author acknowledges that this final version was read and approved. All authors of this article declare that we qualify for authorship as defined by ICMJE http://www.icmje.org/#author. Each author has participated sufficiently in the work and take public responsibility for appropriate portions of the content of this article.

## ETHICS STATEMENT BY ALL AUTHORS:

As this is case report without identifiers, our institution does not require approval from Institutional Review Board (IRB) (or its equivalent)
